# Characterization of the complete plastome sequence of perennial sowthistle, *Sonchus arvensis* (Asteraceae)

**DOI:** 10.1080/23802359.2020.1778569

**Published:** 2020-07-06

**Authors:** Susanna Abdalla Masana, Seon-Hee Kim, Seung-Chul Kim

**Affiliations:** a Department of Biological Sciences, Sungkyunkwan University, Gyeonggi-do, Republic of Korea; b Department of Genetics and Microbiology, Autonomous University of Barcelona, Bellaterra, Spain

**Keywords:** Chloroplast genome, *Sonchus arvensis*, Asteraceae, phylogenetic relationships

## Abstract

The first complete chloroplast genome sequence of *Sonchus arvensis*, a herbaceous perennial, and its phylogenetic position relative to a member of the annual weedy and woody perennial species of *Sonchus* were reported in this study. Here, we assembled the complete plastome sequence of 151,967 base pairs (bp) in length, comprising 84,251 bp of a large single copy (LSC) and 18,184 bp (SSC) of small single copy confined between 24,766 bp of inverted repeats (IR). The genome contained 130 genes, including 87 protein-coding genes, six ribosomal RNA, and 37 transfer RNA genes. The overall GC content was 37.6% (LSC, 35.8%; SSC, 31.5%; IRs, 43.0%). Phylogenetic analysis confirmed that perennial *S. arvensis* is sister to the clade containing the weedy species of *Sonchus*.

*Sonchus arvensis* (milk field thistle, field or perennial sowthistle) belongs to the subgenus *Sonchus*, section *Arvenses*, and represents one of multiple recently radiated and highly unresolved lineages within the genus *Sonchus* (Kim et al. 2004, 2007; Mejías et al. 2018). Two infraspecific taxa, subsp. *uliginosus* (2*n* = 4*x* = 36) and subsp. *arvensis* (2*n* = 6*x* = 54), are currently recognized with native distributions in Europe and western Asia (Boulos 1973). Naturally occurring hybrids produced by the two subspecies have been detected in recently introduced areas where they are sympatric (Lemna and Messersmith 1990). As a noxious weed, *S. arvensis* has been rapidly introduced into various continents and countries, including North America, South America, Australia, Indonesia, Philippines, etc. In addition to being an early successional inhabitant on recently disturbed sites, *S. arvensis* is known for containing several active pharmaceutical compounds (Lemna and Messersmith 1990; Wahyuni et al. 2020). In the case of the phylogenetic position of *S. arvensis*, it is closely related to diploid (2*n* = 2*x* = 18) perennial species of *Sonchus*, including *S. maritimus* and *S. crassifolius* in sect. *Maritimi* and *S. brachyotus* in the same section *Arvenses* (Kim et al. 2007; Mejías et al. 2018). With regard to the plastome organization and evolution within the genus *Sonchus*, we have been generating the complete plastome sequences of woody perennials in the Canary Islands (Cho et al. 2019a), annual weedy species (Cho et al. 2019b), and one paleoendemic perennial species (Kim et al. 2019). Thus, we still know very little about the plastomes of other perennial species of *Sonchus*. As an effort to building a global plastome phylogenomic framework of *Sonchus*, we sequenced the complete plastome sequences of *S. arvensis* and assessed its phylogenetic position.

Total DNA (Voucher specimen: 41°14′27.9″N 83°41′51.1″W, OS418412) was isolated using the DNeasy Plant Mini Kit (Qiagen, Carlsbad, CA) and sequenced by the Illumina HiSeq platform at Macrogen Corporation (Seoul, Korea). A total of 54,961,730 paired-end reads were assembled *de novo* with Velvet v. 1.2.10 using multiple *k*-mers (Zerbino and Birney 2008) and annotated by the Dual Organellar GenoMe Annotator (Wyman et al. 2004), ARAGORN v1.2.36 (Laslett and Canback 2004), and RNAmmer 1.2 Server (Lagesen et al. 2007).

The complete chloroplast genome (GenBank: MT435526) of *S. arvensis* is 151,967 base pairs (bp) in length, containing a large single copy (LSC; 84,251 bp), a small single copy (SSC; 18,184 bp), and two inverted repeats (IRa and IRb; 24,766 bp each). The overall GC content was 37.6% (LSC, 35.8%; SSC, 31.5%; IRs, 43.0%) and the chloroplast contained 130 genes of which 87 were protein-coding genes, six coded for rRNA, and 37 for tRNA genes. To further investigate its phylogenetic position, eight representative species of *Sonchus* were aligned using MAFFT v.7 (Katoh and Standley, 2013) and maximum likelihood (ML) analysis was conducted using IQ-TREE v.1.4.2 (Nguyen et al. 2015). The ML tree showed that *S. arvensis* is sister to a clade containing members of the weedy *Sonchus* species, i.e., *S. oleraceus* and *S. asper* (Figure 1).

**Figure 1.**
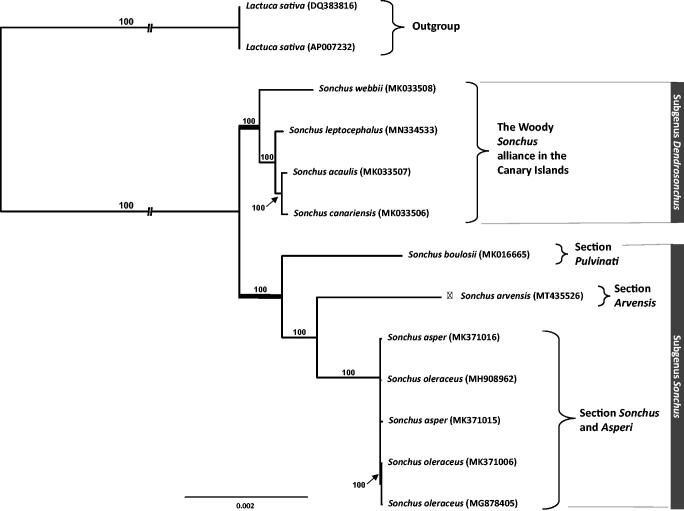
The maximum-likelihood (ML) tree based on 13 representative chloroplast genomes of Asteraceae, 11 of which are from the genus *Sonchus*. The genus *Lactuca* was used as an outgroup, and the bootstrap value based on 1000 replicates is shown in each node.

## Data Availability

The data that support the findings of this study are openly available in GenBank, National Center for for Biotechnology Information at (https://www.ncbi.nlm.nih.gov/genbank/), with reference number of MT435526.
